# Resonant Ionic, Covalent Bond, and Steric Characteristics Present in ^1^Σ_u_^+^ States of Li_2_

**DOI:** 10.3390/molecules27113514

**Published:** 2022-05-30

**Authors:** Michiko Ahn Furudate, Denis Hagebaum-Reignier, Jin-Tae Kim, Gwang-Hi Jeung

**Affiliations:** 1 Department of Mechatronics Engineering, Chungnam National University, Daejeon 34134, Korea; furu@cnu.ac.kr; 2 Aix Marseille Univ, CNRS, Centrale Marseille, iSm2, 13007 Marseille, France; gwang-hi.jeung@univ-amu.fr; 3 Department of Photonic Engineering, Chosun University, Gwangju 61452, Korea

**Keywords:** resonant ionic, ^1^Σ_u_^+^ states of Li_2_, potential energy curves, spectroscopic constants

## Abstract

The molecular bonding in the excited states of the alkali dimers involves the resonant ionic, covalent bond and steric interactions. We show here the case of the ^1^Σ_u_^+^ states of Li_2_ by ab initio calculation. These interactions as functions of the internuclear distance lead to complex potential energy curves, providing an important application for high resolution laser spectroscopy. The spectroscopic constants for the 4 and 5 ^1^Σ_u_^+^ states are obtained for the first time.

## 1. Introduction

The molecular bonding between atoms is classified according to its characteristics into three main types of bonds; namely, the covalent bonding, ionic bonding, and Van der Waals bonding. Former two types are present for relatively short internuclear distances and there also exists intermediate type of bonding that has both partially covalent and partially ionic characters. In contrast, the Van der Waals type is concerned for larger distances and is often disqualified as chemical bonding but is counted as a physical bonding. In the case of homopolar dimers, another type of ionic bonding exists, namely the resonant ionic bonding, where the time-averaged dipole moment is zero but the instantaneous one is non-zero [1].

Colliding atoms can also repulse each other without making a chemical bonding due to the steric repulsion as in the example of the rare gas atoms or between closed-shell molecules (N_2_, O_2_, etc.). The steric repulsion is strong enough at room temperature to impede the formation of Van der Waals complex. In the case of the excited states of Rydberg type, the steric repulsion concerns an interaction between a valence orbital and a Rydberg orbital. As the latter has a diffuse electron distribution, the repulsion is much weaker than between the valence orbitals. However, the strength of the repulsion decreases slowly for large internuclear distance and shows undulating property, as was shown in some diatomics [2].

Some experimental and theoretical studies on highly excited electronic states of Li_2_ of Rydberg character have been reported [3,4,5,6,7,8,9,10,11,12,13,14]. Such states were also used to probe lower-lying states [14]. As has been commented in those works, it is difficult to find the correlation between the molecular states and the atomic asymptotes as many more molecular states can be made from a given atomic states and many avoided crossings occur due to the high density of states. The energy levels of the molecular states are also difficult to anticipate as the molecular bond energies differ much according to the point group symmetry and also depend on the atomic orbitals involved. The Rydberg character has been initially defined for the atomic states as one made from the principal quantum number greater than that of the ground state. In forming a diatomic molecule, the atomic orbitals from both atoms are all mixed up resulting in a difficulty of defining the Rydberg character. In the case of Li_2_, there are also many states made from (2p + 2p) that interact with other states. This can be seen from a recent theoretical calculation of some Rydberg states [14] where subtleties related to deriving quantum defects were presented. It is apparent that no simple model without complication can give molecular energy levels. As a consequence, it is not evident to identify the atomic asymptote for an unknown electronic state that lies far above the ground state.

In the excited states, as the electronic energy is much higher than in the ground state, it is easier for the electron to be ionized. It makes the resonant ionic state much more probable, where two forms, Li^+^/Li^−^ and Li^−^/Li^+^, have the equal probability to exist. Due to the symmetry reason, this can happen for the ^1^Σ^+^ states in Li_2_. The resonant ionic state is diabatically correlated to the asymptotic energy that is the difference between the ionization potential (IP) and the electronic affinity (EA) of the Li atom, IP−EA. On the first approximation, the potential energy as a function of the internuclear distance (R), i.e., potential energy curve (PEC), of such resonant ionic state would be IP−EA−1/R + SR, where SR is the steric repulsion term between the two cores in the 1s^2^ state of the Li atom.

Among these excited states, the second singlet state of Σ_u_^+^ symmetry, hereafter named 2 ^1^Σ_u_^+^, has attracted attention of both experimentalists and theoreticians due to the presence of two wells in its potential curve. This unusual potential shape was already predicted theoretically in the early 1980′s by Konowalow and Fish effective core potential calculation [3]. Further high-resolution spectroscopic studies showed the survival of up to eight vibrational states in the inner well and some vibrational states of the outer well could also be detected due to tunneling effects [8]. The present work revisits the origin of the double-well potential of the 2^1^Σ_u_^+^ and presents more general properties of the excited electronic states that this state appears to share. We also give here the spectroscopic constants for the 4 and 5 ^1^Σ_u_^+^ states for the first time that could be a good guide for the precision spectroscopic measurements in energy in future.

## 2. Method of Computation

The basis set used in this work includes 13s8p5d3f Gaussian atomic basis functions. It was taken from the 15s10p6d3f functions previously described [2]. This basis was specially made to well represent high-lying excited states up to 6s + 2s asymptotic state of Li_2_.

In this basis set, the most diffuse two s-type functions cause the linear dependence problem for short internuclear distance, resulting from the large overlap integrals between such diffuse s-type functions. So, we had to use different basis sets, with and without these two, to compare the resulting energy (see below in this section). The most diffuse two p-type functions and 1 d-type functions were also tested with and without, as the atomic asymptotes we report here, (3s + 2s), (3p + 2s) and (3d + 2s), are very well described with the truncated basis set. We have used MOLPRO [15] program packages for the molecular calculation. In the multi-configuration (MC) self-consistent-field (SCF) calculations, we have used four σ_g_, four σ_u_, one π_g_, one π_u_ molecular orbitals (Mos) in the valence active space. In the multi-reference (MR) configuration interaction (CI) calculations, we have used all single and double excitations from the MC-SCF active space. The four core electrons were also allowed to make all possible single and double substitutions. In all, 250,549 configuration state functions (CSFs) were generated for the ^1^Σ_u_^+^ states.

Transition dipole moments from n^1^Σ_u_^+^ states to the ground state X^1^Σ_g_^+^ are also calculated by MOLPRO. To calculate the PEC of the ground state, we have used the basis set and the active space consistent with those for n ^1^Σ_u_^+^ mentioned above.

From computed potential energy curves for n ^1^Σ_u_^+^ states, we have determined vibrational levels for each electronic state by using the LEVEL16 program [16]. This program solves the radial Schrodinger equation for an input PEC, and outputs bound and/or quasi-bound levels, and eigenfunctions and various constants for those levels.

## 3. Results and Discussion

**Figure 1:**
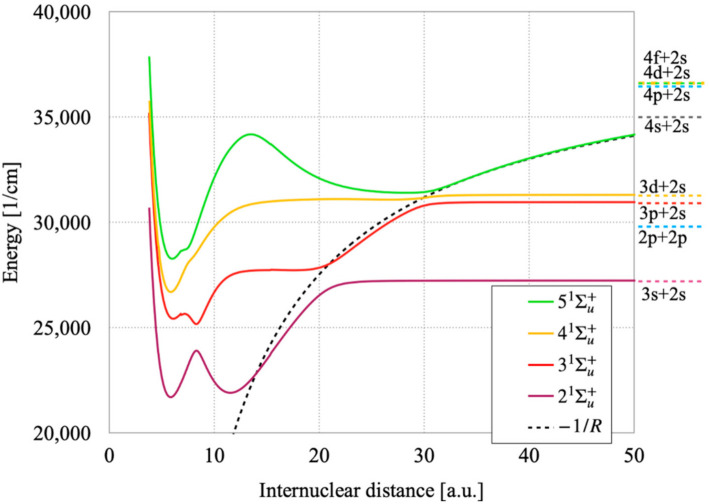
Potential energy curves of the 2, 3, 4 and 5 ^1^Σ_u_^+^ states of ^7^Li_2_.

The PECs for the second, third, fourth, and fifth ^1^Σ_u_^+^ states are reported in Figure 1. The energy scale in these figures is with respect to the dissociation limit of the ground state (2s + 2s). To obtain the transition energy from the ground state molecule one has to add the ground state dissociation energy (*D*_e_), 8517 cm^–1^ [17]. Here, *T_e_* is the potential energy difference between the minimum of the ground state and the minimum of the excited state. That should not be confounded with *T_0_*, which is the potential energy difference between the lowest vibrational level of the ground electronic state and the lowest vibrational level of the excited electronic state. We can see here that the second state (2 ^1^Σ_u_^+^) has two potential wells. The inner well of 2 ^1^Σ_u_^+^ is made from the covalent bonding of (3d + 2s) while the outer well is made from (3s + 2s). The 3dσ atomic orbital (AO) is more directed along the internuclear axis, so it can make more efficient bonding with 2s AO in comparison with much more diffuse 3s AO. As a result, the bond strength of (3d + 2s) is much larger than (3s + 2s). In the intermediate distance between these two, from around 10 Bohr to the bottom of the outer well, 2p AO takes part in the bonding, corresponding to the repulsive part of (2p + 2s) ^1^Σ_u_^+^. All those changes result in the avoided crossing between the two potential wells, thus making atypical PEC with double well. This state becomes resonant ionic at around 20 Bohr and this part of PEC is a result of the avoided crossing between the covalent (3s + 2s) bond and the resonant ionic (Li^+^/Li^−^ and Li^−^/Li^+^) bond.

In the case of the third state (3 ^1^Σ_u_^+^), three kinds of bonds, (3s + 2s), (3p + 2s) and (3d + 2s), interfere to make a very strange PEC with double well. An avoided crossing between (3p + 2s) and the resonant ionic bonds occurs around 30 Bohr.

Table 1 shows the critical points, potential well (maxima, M), potential barrier (minima, m) and inflection points (i), resulted in two different basis sets, one with the two diffuse s-type Gaussian functions, and another one without them. We can see here that the differences in distance and relative energy are very small for the 2 ^1^Σ_u_^+^ and 3 ^1^Σ_u_^+^ states. For the 4 ^1^Σ_u_^+^ state, however, the potential well depth (m) differs by about 200 cm^−1^. We have connected PECs from this smaller basis set (13s8p5d3f) to the corresponding PECs from the full basis (15s10p6d3f) at the interatomic distance of 5.3 Bohr. To make smooth connections, each PEC from the smaller basis set is shifted to have the same minimum energy values at the same interatomic distance as the corresponding state PECs from the full basis set.

The fourth state (4 ^1^Σ_u_^+^) has a single well perturbed by the upper lying states, at the outer part of the well around 8 Bohr, as can be seen in the inflection points in Table 1. The fifth state (4 ^1^Σ_u_^+^) shows a potential barrier around the maximum at 13.5 Bohr. It is due to an avoided crossing between the repulsive 3d + 2s and higher lying state.

**Figure 2:**
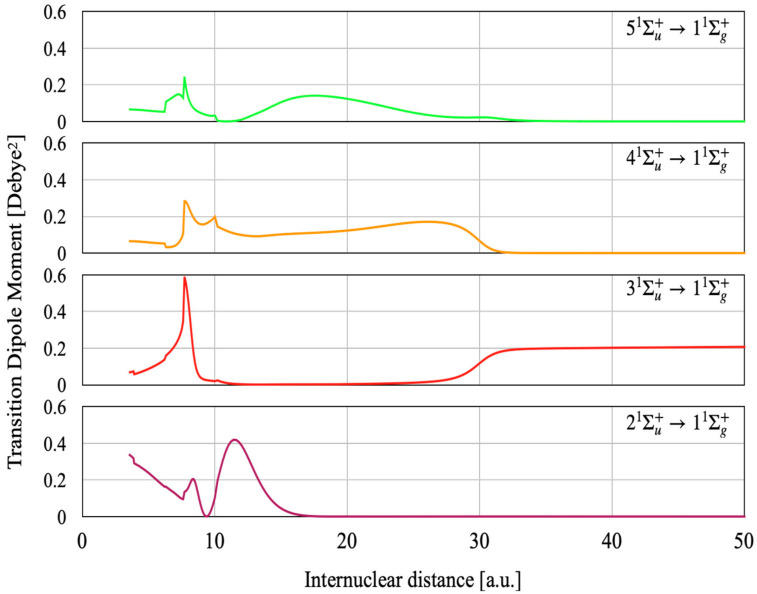
Transitional dipole moment (Squared) from the ground state to the ^1^Σ_g_^+^ states.

The transition dipole moments (TDMs) from the ground state to these ^1^Σ_u_^+^ states as functions of the internuclear distance (R) are shown in Figure 2. We can better understand the changing molecular electronic eigenfunctions with respect to R by comparing Figure 1 and Figure 2. For the 2 ^1^Σ_u_^+^ state, the TDM goes to zero at around 9 Bohr where 3d AO is predominant, as the 3d-2s transition is dipole forbidden. One can also see a large TDM around 12 Bohr where the 2p AO takes part in the bonding. For larger distances, the TDM rapidly vanishes where the resonant ionic configuration becomes predominant then the asymptotic neutral configuration (3s + 2s) replaces the former.

For the 2^1^Σ_u_^+^ state, there is a perfect concordance between the PEC and TDM around R = 15 Bohr, where the resonant ionic configuration relays the mixed valence configuration. Indeed, the TDM decreases to zero for R between 15 and 20 Bohr as the 2^1^Σ_u_^+^ state is resonant ionic. For the resonant ionic state, each atom can be described as 2s^2^/2 and the (2s^2^/2) -2s transition is dipole forbidden. For R > 20 Bohr, the TDM remains zero as the 2 ^1^Σ_u_^+^ state becomes 3s + 2s, the 3s-2s being forbidden. The TDM of the 3^1^Σ_u_^+^ state remains zero for a large domain of R values because this state correlates to the 3s + 2s dissociation asymptote for R < 20 Bohr. The 3 ^1^Σ_u_^+^ state becomes resonant ionic at around 20 Bohr, so its TDM remains zero between 20 and 30 Bohr. For R > 30 Bohr, this state becomes 3p + 2s and its TDM converges to the 3p-2s value. The TDM of 4 ^1^Σ_u_^+^ converges to zero for R > 0 Bohr, as it corresponds to the 3d-2s atomic transition that is dipole forbidden. The TDM of the 5 ^1^Σ_u_^+^ state converges to zero for R > 32 Bohr as it corresponds to resonant ionic part. The 5 ^1^Σ_u_^+^ state dissociates into 4s + 2s, so its TDM remains zero for R corresponding to dissociation.

**Figure 3:**
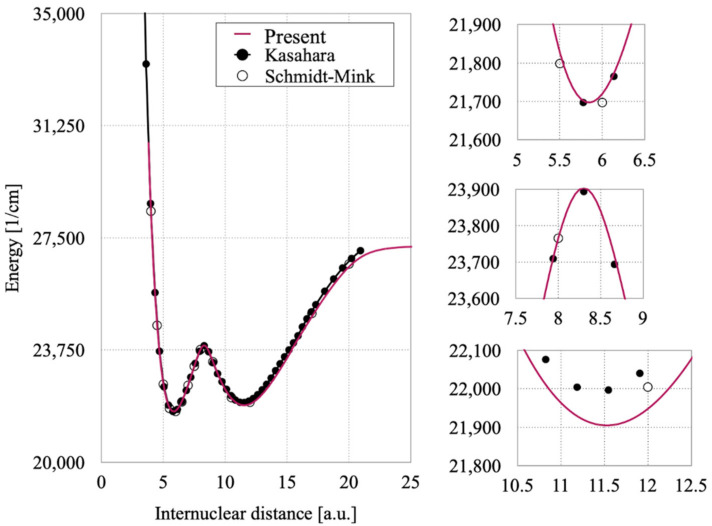
Comparisons of the potential curves of the 2 ^1^Σ_u_^+^ state between the present work and previously published results (**left**) with three close-up figures (**right**).

**Figure 4:**
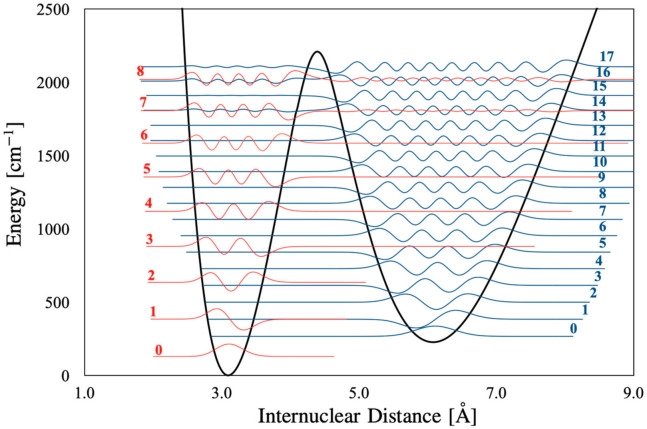
The eigenfunctions of vibrational levels *v* = 0–16 (*v*_i_ = 0–8 and *v*_o_ = 0–17) for 2^1^Σ_u_^+^ (J = 0). The amplitudes are magnified by a factor of 50.

The vibrational levels obtained from the LEVEL16 program for 2 ^1^Σ_u_ state are summarized in Table 2 and compared with Kasahara’s experimental and semi-classical results [8]. As shown in Figure 3 Kasahara’s potential curve in the inner well and the barrier regions matches well with one calculated by us. The lowest three levels in the inner well are in good agreement with Kasahara’s values; the error is within the spectroscopic accuracy. As the level increase, the error increases up to about 8 cm^−1^. For the outer well, we made the correspondence of the outer well vibrational levels in two works considering the relations of vibrational levels in the inner and outer wells from the plot of wave functions in Kasahara’s work [8] and the present work (Figure 4). The upper vibrational levels in the present study agree reasonably with Kasahara’s values and the differences within 9 cm^−1^. However, the difference is significant in the lower levels. Our outer potential well is lower than that of Kasahara’s by about between 59 cm^−1^ and 81 cm^−1^. With the increases of internuclear distance the differences are becoming larger. This disparity may be originated from the adjustment made by Kasahara et al. Indeed, they have obtained the vibrational levels from the PEC optimized to fit their experimental term values by the inverted perturbation approach (IPA) [18]. As the initial outer PEC for the IPA, they used the theoretical PEC of Schmidt-Mink [4], and the curve was shifted up by about 35 cm^−1^ to roughly reproduce the experimentally determined vibrational levels near the local maximum. Since experimental data to optimize the outer well is available only for a few levels near the local maximum, the accuracy of the PEC for the lower part of the outer well would be less reliable, as they have mentioned in their work.

Our spectroscopic constants such as *R*_e_*, T*_e_*, B*_e_*, α*_e_*, ω*_e_*, ω*_e_*x*_e_ of the inner and outer wells of the 2 ^1^Σ_u_^+^ state as shown in Table 3 are closer to Musiał et al.’s experimental ones. Spectroscopic constant T_e_ in other publications gave a large difference of around 300 cm^−1^, as can be seen in Table 3, although all of previous reported constants except α_e_ agree within ~1%. Our spectroscopic constants for the inner and outer wells of the 3 ^1^Σ_u_^+^ state as shown in Table 3 are also closer to Musiał et al.’s experimental ones. In Table 4, one can see that our spectroscopic constants fitted for the 2 ^1^Σ_u_^+^ state using Dunham polynomials match well with those of Kasahara et al.

We have new spectroscopic constants for the 4 ^1^Σ_u_^+^ and 5 ^1^Σ_u_^+^ states in Table 4 that could serve as a guide to the future experimental observation using far UV photons with short wavelengths. Especially, the 5 ^1^Σ_u_^+^ state has a wide potential barrier so that the experimental observation of this state will be a challenging task.

**Figure 5:**
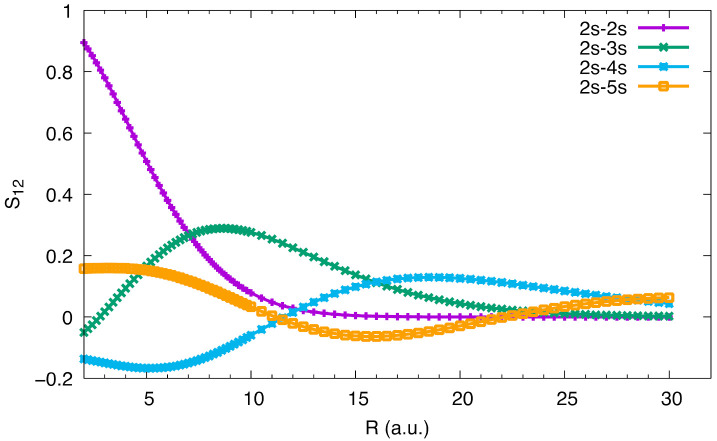
Overlap integrals between ns and 2s atomic orbitals as functions of R.

There is one peculiarity in the PEC of the 5 ^1^Σ_u_^+^ state. This state should be converging to the 3s + 2s asymptote before meeting the resonant ionic state at large distance (at about 25 Bohr). So one might expect the PEC from about 10 Bohr to 25 Bohr to remain below the 3s + 2s asymptote. However, it is not the case. In fact, the potential energy between 10.8 Bohr to 17.4 Bohr (see Table 1) remains significantly higher than the asymptote, by 478 cm^–1^ at the top of the barrier according to our calculation. We think this may be caused by the steric repulsion between 3s and 2s atomic orbitals (AOs). Figure 5 shows the overlap integrals between 2s/2s, 3s/2s, 4s/2s and 5s/2s. The overlap between 2s/2s increases as the internuclear distance (R) becomes small, while that of 3s/2s has one maximum, 4s/2s two maxima, and 5s/2s three maxima, due to the nodal structure of the AO. For a given R, the magnitude of the overlap decreases rapidly in the order of 2s/2s/, 3s/2s, 4s/2s and 5s/2s, as the amplitude of excited AOs decrease according to the degree of excitation. The steric repulsion is approximately a function of the amplitude of the overlap integral, so the former behaves similar to the latter.

As other effects, in particular the reorganization of the electron distribution in forming the molecule due to the presence of other core (1s^2^), the correlation effect between the two electrons, intervene apart from the steric repulsion, it is not easy to attribute one contribution for the peculiarity mentioned above. In the case where the interatomic bonding is not strong, one can better see the oscillating PEC as has been reported before [2,19]. There are other states, e.g., the excited ^1^Σ_g_^+^ states of Li_2_ and in other alkali dimers, that show the same aspect.

## 4. Conclusions

The potential energy curves (PECs) for the second, third, fourth, and fifth ^1^Σ_u_^+^ states calculated in this work show all essential features of the excited electronic states: covalent contribution, resonant ionic contribution and the steric contribution. These three contributions appear in different domains of the internuclear distance and in different levels of the potential energy. As the excited atomic orbitals becomes more diffuse as the degree of excitation increases, all three contributions extend to larger nuclear distance. The avoided crossings for the highly excited states appearing at relatively short internuclear distances are caused by the interaction between different components of the covalent bonding, attractive and repulsive. As the resonant ionic contribution behaves approximately according to −1/R, the perturbation caused by this contribution appears at larger distances as the degree of electronic excitation increases. The steric repulsion shows undulating behavior due to the radial part of the atomic orbital density for the excited state, and that is reflected at large internuclear distance. The case of the ^1^Σ_u_^+^ states shown in this work illustrate very well the complexity of the potential energy curves and the nature of electronic functions. New spectroscopic constants for the 4 and 5 ^1^Σ_u_^+^ states as well as transition dipole moments from the ground state to the ^1^Σ_u_^+^ states as functions of the internuclear distance are reported in this work for the first time, too.

## Figures and Tables

**Table 1:** Critical points (maxima M, minima m, inflection i, asymptote a), of the electronic states calculated in this work.

Basis		15s10p6d3f		13s8p5d3f	
States		R (a.u)	E (cm^−1^)	R (a.u)	E (cm^−1^)
5 ^1^Σ_u_^+^	m	6.00	28,271	6.000	28,298
	i	6.925	28,657		
	i	7.175	28,702		
	i	8.525	30,098		
	M	13.50	34,177		
	i	16.125	33,428		
	m	28.35	31,404		
	i	33.30	31,924		
	*a*	100	35,030		
	*a* (ex)	∞ (4s + 2s)	35,012		
4 ^1^Σ_u_^+^	m	5.849	26,693	5.830	26,885
	i	7.025	27,595		
	i	7.775	28,236		
	i	8.725	28,891		
	*a*	100	31,302		
	*a* (ex)	∞ (3d + 2s)	31,283		
3 ^1^Σ_u_^+^	m	6.091	25,424	6.099	25,439
	i	6.925	25,622		
	M	7.184	25,652		
	i	7.875	25,359		
	m	8.281	25,170	8.311	25,251
	i	9.075	25,779		
	i	14.95	27,727		
	M	15.40	27,748		
	i	16.00	27,740		
	m	17.55	27,723		
	i	23.35	28,834		
	*a*	100	30,961		
	*a* (ex)	∞ (3p + 2s)	30,925		
2 ^1^Σ_u_^+^	m	5.844	21,697	5.843	21,702
	i	7.375	23,039		
	M	8.300	23,904		
	i	9.025	23,346		
	m	11.526	21,904	11.505	21,937
	i	15.725	23,976		
	*a*	100	27,237		
	*a* (ex)	∞ (3s + 2s)	27,206		

**Table 2:** Vibrational energy levels of 2 ^1^Σ_u_^+^ (J = 0) of ^7^Li_2_.

2^1^Σ_u_^+^ (J = 0) Inner Well							
*G_v_* (cm^−1^)					*B_v_* (cm^−1^)		
*v_i_*	Present	*v_i_*	Kasahara	Present-Kasahara	Present	Kasahara	Present-Kasahara
**0**	129.0231	**0**	129.0751	−0.0520	0.49949	0.49865	0.00084
**1**	384.0727	**1**	384.1343	−0.0616	0.49330	0.49232	0.00098
**2**	634.7832	**2**	634.9956	−0.2124	0.48671	0.48587	0.00084
**3**	880.0632	**3**	881.4854	−1.4222	0.47933	0.47924	0.00009
**4**	1119.3332	**4**	1123.3140	−3.9808	0.47200	0.47231	−0.00031
**5**	1354.0883	**5**	1359.9908	−5.9025	0.46547	0.46490	0.00057
**6**	1583.9713	**6**	1590.6952	−6.7239	0.45733	0.45670	0.00063
**7**	1806.1984	**7**	1814.0597	−7.8613	0.43857	0.44691	−0.00834
**8**	2019.1734	**8**	2026.9763	−7.8029	0.39926	0.38556	0.01370
**2^1^Σ_u_^+^ (J = 0) Outer Well**							
***G_v_* (cm^−1^)**					***B_v_* (cm^−1^)**		
***v_o_***	**Present**	***v_o_***	**Kasahara**	**Present-Kasahara**	**Present**	**Kasahara**	**Present-Kasahara**
**0**	266.2942		-	-	0.12910	-	-
**1**	383.2602	**0**	361.8034	21.4568	0.12901	0.13129	−0.00125
**2**	499.2858	**1**	483.0413	16.2445	0.12891	0.13061	−0.00131
**3**	614.2992	**2**	603.7395	10.5597	0.12879	0.13016	−0.00153
**4**	728.3109	**3**	722.3377	5.9732	0.12868	0.13010	−0.00180
**5**	841.2961	**4**	839.1467	2.1494	0.12856	0.13021	−0.00248
**6**	953.2471	**5**	953.5825	−0.3354	0.12846	0.13036	−0.00349
**7**	1064.1449	**6**	1065.6593	−1.5144	0.12836	0.13094	−0.00427
**8**	1173.9686	**7**	1175.8812	−1.9126	0.12829	0.13185	−0.00433
**9**	1282.6964	**8**	1285.0167	−2.3203	0.12825	0.13256	−0.00362
**10**	1390.3004	**9**	1393.7730	−3.4726	0.12826	0.13258	−0.00272
**11**	1496.7460	**10**	1502.0048	−5.2588	0.12832	0.13188	−0.00237
**12**	1601.9835	**11**	1608.7631	−6.7796	0.12848	0.13104	−0.01001
**13**	1705.9445	**12**	1713.5840	−7.6395	0.12878	0.13085	0.00548
**14**	1808.6007	**13**	1816.8750	−8.2743	0.13818	0.13879	−0.04630
**15**	1909.5915	**14**	1918.2592	−8.6677	0.13042	0.13270	0.02033
**16**	2007.7026	**15**	2015.4642	−7.7616	0.16340	0.17672	−0.08919
**17**	2105.6652	**16**	2112.3711	−6.7059	0.14045	0.14307	−0.00160
		**17**	2193.0165	-	-	0.22964	-

**Table 3:** Spectroscopic constants of 2, 3, 4 and 5 ^1^Σ_u_^+^ states of ^7^Li_2_.

**State**		***R*_e_**	***D*_e_**	***T*_e_**	***B*_e_**	***α*_e_**	***ω*_e_**	***ω*_e_ *x*_e_**
		**(Å)**	**(cm^−1^)**	**(cm^−1^)**	**(cm^−1^)**	**(cm^−1^)**	**(cm^−1^)**	**(cm^−1^)**
2^1^Σ_u_^+^ Inner	Present	3.092	5540	29,986.74	0.502	0.0052	258.7	1.60
	Kim [14]	3.078	5702.6	30,144.3	0.507	0.0062	259.7	2.01
	Lee [12]	3.125	5568	29,653				
	Musial [13]	3.093	5608	30,044			260	
	Jasik [9]	3.081	5674.0	30,094.0			259.82	
	Linton [5]	3.094		30,100.3	0.502	0.0066	259.9	2.23
	Exp. [8]	3.096		30,101.407	0.502	0.0062	259.003	1.71
2^1^Σ_u_^+^ Outer	Present	6.100	5333	30,194.00	0.129	4.978 × 10^−5^	117.9	0.4579
	Lee [12]	6.072	5367	29,853				
	Musial [13]	6.088	5389	30,261			118	
	Jasik [9]	6.072	5413	30,285			119.05	
	Exp. [8]	6.037		30,400.137				
3^1^Σ_u_^+^ Inner	Present	3.223	5537	33,713.25	0.465	0.0248	188.9	-
	Lee [12]	3.288	5663	33,300				
	Musial [13]	3.250	5628	33,762			193	
3^1^Σ_u_^+^ Outer	Present	4.382	5791	33,460.00	0.249	−0.0033	272.9	-
	Kim [14]	4.351	5894	33,669.8			304.0	
	Lee [12]	4.384	5872	33,091			306	
	Musial [13]	4.360	5874	33,512				
	Jasik [9]	4.369	5854	33,668.0			295.3	
4^1^Σ_u_^+^	Present	3.095	4663	34,982.61	0.4993	0.00250	262.4	6.6591
5^1^Σ_u_^+^	Present	3.173	6759	36,554.79	0.4775	0.0106	228.6	2.4409

**Table 4:** Spectroscopic constants of 2, 3, 4 and 5 ^1^Σ_u_^+^ states of ^7^Li_2_ for Dunham expression.

State	2^1^Σ_u_^+^ Inner		2^1^Σ_u_^+^ Outer
	Present	Kasahara	Present
*v*_max_	4		13
*j*_max_	6		6
Err_max_	5.789 × 10^−2^		4.336 × 10^−2^
RMS	4.898 × 10^−3^		2.794 × 10^−3^
Y_00_	0.04751	0.1101	207.5
Y_10_	258.762	259.003	117.9
Y_20_	−1.606	−1.7124	−0.4579
Y_30_	−1.386 × 10^−1^	−1.811 × 10^−1^	−3.774 × 10^−3^
Y_01_	0.5031	0.5016	0.1289
Y_11_	−6.896 × 10^−3^	−6.247 × 10^−3^	−4.9781 × 10^−5^

## References

[1] Jeung G.-H., Ross A.J. (1988). Electronic structure of the lowest ^1,3^Σ_g_^+^, ^1,3^Σ_u_^+^, ^1,3^Π_g,_^1,3^Π_u_, ^1,3^Δ_g_ and ^1,3^Δ_u_ states of K_2_ from valence CI calculations. J. Phys. B At. Mol. Opt. Phys..

[2] Yiannopoulou A., Jeung G.-H., Park S.J., Lee H.S., Lee Y.S. (1999). Undulations of the potential-energy curves for highly excited electronic states in diatomic molecules related to the atomic orbital undulations. Phys. Rev. A.

[3] Konowalow D.D., Fish J.L. (1984). The molecular electronic structure of the twenty-six lowest lying states of Li_2_ at short and intermediate internuclear separations. Chem. Phys..

[4] Schmidt-Mink I., Müller W., Meyer W. (1985). Ground- and excited-state properties of Li_2_ and Li_2_^+^ from ab initio calculations with effective core polarization potentials. Chem. Phys..

[5] Linton C., Martin F., Bacis R., Vergès J. (1989). Observation and analysis of the 2 ^1^Σ_u_^+^ “double minimum” state of Li_2_. J. Mol. Spectrosc..

[6] Poteau R., Spiegelmann F. (1995). Calculation of the Electronic Spectrum of Li_2_ Using Effective Core Pseudopotentials and l -Dependent Core Polarization Potentials. J. Mol. Spectrosc..

[7] Ross A.J., Crozet P., Linton C., Martin F., Russier I., Yiannopoulou A. (1998). On the 5d^1^Π_g_→ 2^1^Σ^+^_u_ and 5d^1^Π_g_→C^1^Π_u_Fluorescence in ^7^Li_2_. J. Mol. Spectrosc..

[8] Kasahara S., Kowalczyk P., Kabir H., Baba M., Katô H. (2000). Doppler-free UV-visible optical–optical double resonance polarization spectroscopy of the 2 ^1^Σ_u_^+^ double minimum state and the C^1^Π_u_ state of Li_2_. J. Chem. Phys..

[9] Jasik P., Sienkiewicz J.E. (2006). Calculation of adiabatic potentials of Li_2_. Chem. Phys..

[10] Jędrzejewski-Szmek Z., Grochola A., Jastrzebski W., Kowalczyk P. (2007). The 5^1^Π_u_ electronic state of the lithium dimer. Chem. Phys. Lett..

[11] Grochola A., Kowalczyk P., Jastrzebski W. (2010). Rydberg states of the Li_2_ molecule studied by polarization labelling spectroscopy. J. Phys. B At. Mol. Opt. Phys..

[12] Lee C.-W. (2014). Study of the Valence and Rydberg States of a Lithium Dimer by the Multi-reference Configuration-interaction Method, *Bull*. Korean Chem. Soc..

[13] Musiał M., Kucharski S.A. (2014). First Principle Calculations of the Potential Energy Curves for Electronic States of the Lithium Dimer. J. Chem. Theory Comput..

[14] Kim H.-J., Lee C.-W. (2015). Rydberg series of ^1^Σ_u_^+^ and ^1^Δ_u_ states of the Li_2_ molecule studied by the promotion model. J. Phys. B At. Mol. Opt. Phys..

[15] Werner H.-J., Knowles P.J., Knizia G., Manby F.R., Schütz M. (2012). Molpro: A general-purpose quantum chemistry program package. WIREs Comput. Mol. Sci..

[16] Le Roy R.J. (2017). LEVEL: A computer program for solving the radial Schrödinger equation for bound and quasibound levels. J. Quant. Spectrosc. Radiat. Transf..

[17] Coxon J.A., Melville T.C. (2006). Application of direct potential fitting to line position data for the X^1^Σ_g_^+^ and A^1^Σ_u_^+^ states of Li_2_. J. Mol. Spectrosc..

[18] Kosman W.M., Hinze J. (1975). Inverse perturbation analysis: Improving the accuracy of potential energy curves. J. Mol. Spectrosc..

[19] Geum N., Jeung G.-H. (2001). Undulating potential curves of the Rydberg states of NaHe. Chem. Phys. Lett..

